# The dynamics of smallholder farmers’ acquisition and distribution of sweetpotato vines in the Lake Victoria Zone Region, Tanzania

**DOI:** 10.1007/s12571-018-0776-5

**Published:** 2018-03-20

**Authors:** Rahma I. Adam, Lone Badstue, Kirimi Sindi

**Affiliations:** 1 Agricultural Economics, Sociology and Education, The Pennsylvania State University, State College, PA 16802, USA; 2 Present address: International Maize and Wheat Improvement Center (CIMMYT), c/o The World Agroforestry Center, ICRAF House, United Nations Avenue, Gigiri, P.O. Box 1041, Nairobi 0062, Kenya; 3 Hellen Keller International, Nairobi, Kenya; 4 Present address: International Maize and Wheat Improvement Center (CIMMYT), Km. 45, Carretera Mexico-Veracruz, El Batan, CP 56130 Texcoco, Edo. De Mexico, Mexico; 5 International Potato Center (CIP), Kacyiru Road St. 563, Plot No. 1490- Gasabo District, Kigali, Rwanda

**Keywords:** Smallholder agriculture, Informal seed system, Vine access, Tanzania

## Abstract

This paper offers new insights into smallholder farmer’s practices regarding acquisition and distribution of sweetpotato planting material in the Mwanza and Mara regions of Tanzania by examining three specific issues: (i) farmers’ sources of planting material; (ii) factors that influence farmers’ sourcing of planting materials outside their own farms and (iii) the types of transactions and social relations involved in farmers’ acquisition and distribution of sweetpotato planting material. Data were collected using mixed methods, including a survey of 621 households across nine districts, semi-structured key informant interviews with 28 women sweetpotato farmers, and six focus group discussions. Findings show that farmers in the study area rely almost exclusively on informal seed systems, and that the majority (> 56%) produce their own planting material. Individual, household and community level factors influence farmers’ acquisition of planting materials outside their own farms. The sources and mode of transaction related to acquisition/distribution of planting material are strongly influenced by the type of social relationship between the parties involved. Strong social ties facilitate the majority of local planting material acquisitions/distributions, and favor provision of locally available planting material as a gift/without payment.Weak social ties are primarily associated with the transaction modality of purchase/sale, and frequently help facilitate acquisition of new or exotic planting material. The findings provide entry points both for entities that seek to enhance small-scale farmers’ access to improved, high quality sweetpotato germplasm, as well as broader efforts to strengthen research and development strategies for integrating formal and informal seed systems.

## 1 Introduction

The problems of hunger and malnutrition are widespread in rural Sub-Saharan Africa (SSA), and ensuring food security is a major development priority in the region. The bulk of food crop producers in SSA are smallholder farmers who have become the center of attention for development actors seeking to promote food security. In Tanzania, for example, smallholder farmers are supported by a range of actors to improve their household food security through the adoption of different sweetpotato (*Ipomea batatas* (L.) Lam) varieties. In the *Marando Bora* project (or *Quality Vines* project in Kiswahili),^1^ the International Potato Center (CIP) and Catholic Relief Services (CRS) together with local governmental (GOs) and non-governmental organizations (NGOs) provided farmers with quality planting material of improved high-yielding sweetpotato varieties. Based in the Lake Region of Tanzania, *Marando Bora* addressed issues associated with the availability and distribution of sweetpotato planting material, i.e., vine cuttings, by developing a sustainable “seed system”^2^ for sweetpotato.

The project’s ultimate aim was to improve the food and nutrition security of subsistence farmers who rely on sweetpotato as a staple food and to increase the incomes of those who produce and sell vine cuttings or sweetpotato roots. By establishing a network of decentralized vine multipliers (DVMs) in the region, the project sought to ensure timely access to virus-free, quality planting material of improved sweetpotato varieties at the beginning of the rain season. DVMs were designed to be the residual sources of quality planting material in the project intervention areas.

Sweetpotato can be produced through vegetative propagation either using the roots, or more typically, by the cutting and replanting of vine segments. An energy-dense food (Kapinga et al. 1995), sweetpotato matures fast and can grow under harsh and stressful conditions (Wolfe 1992). Thus, during periods of food scarcity, it can play a critical role in complementing other food crops and serving as a famine reserve when cereal crops fail (Namanda et al. 2012).

A major challenge preventing the widespread production of vegetatively propagated crops (VPCs), including sweetpotatoes by smallholders in rural Africa, is the limited production of and access to good quality planting materials. From his research in Uganda, Gibson (2013) identified three systems for the distribution of sweetpotato varieties: (1) formal, (2) project-based and (3) informal. Describing the limitations of each system, he explains that the formal system lacks capacity to distribute released varieties, the project based system lacks sustainability and the informal system lacks access to improved varieties.

Although often neglected in research and project interventions, the informal system continues to dominate sweetpotato production by smallholders in most parts of rural Africa. McGuire and Sperling (2016) used data of Seed System Security Assessments (SSSAs) collected between 2009 and 2012 in six countries: Malawi, Kenya, Democratic Republic of Congo, South Sudan, Zimbabwe and Haiti to show the source of farmer planting material for the most recent season, clustering by crop across sites. Analyzing a range of crops (cereals, legumes and VPCs), including sweetpotato, findings revealed that (i) 79.2% of sweetpotato cuttings were obtained from farmers’ own stocks, (ii) 14.6% from friends, neighbors and relatives, (iii) 3.5% from the local market and (iv) 2.7% from NGOs.

From a seed acquisition perspective, farmers’ informal social networks are critical for VPCs, such as cassava (*Manihot esculenta Crantz*), banana (*Musa acuminata Colla*), sweetpotato and Irish potatoes (*Solanum tuberosum* L.), because formal market options remain limited in many countries (McGuire and Sperling 2016). Apart from producing one’s own sweetpotato vines for planting, in many cases farmers’ informal networks are often the only other source of planting material (Coomes et al. 2015). Indeed, according to Sperling et al. (2013), VPCs’ formal seed system provides just one tenth of planting material. A key issue is that commercial seed companies tend to produce less planting material for VPCs, such as sweetpotato and cassava, which are deemed unprofitable (Moyo et al. 2004) and often lack quality control arrangements for VPCs able to operate at scale.

Rather than build on existing informal systems for accessing sweetpotato planting material, the project-based system (applied in both Uganda and Tanzanian settings) has often created and operated through new groups of DVMs or farmer field schools (Stathers et al. 2005, 2013), whose produce is typically purchased by NGO or government contracts and distributed free to farmers. However, as Gibson (2013) points out, the project-based model has in many cases been unable to sustain itself after donors end their financial and technical support.

By contrast, a robust informal sweetpotato seed system (vines) exists in the Gulu region of Northern Uganda, where research conducted from 2013 to 2015 revealed a diverse set of actors, including local vine multipliers, traders, dry season root farmers, transporters and town sellers, all engaged in a vibrant marketing system of vines (Rachkara et al. 2017).

The contributions and potential for improving the distribution of good quality planting material by seed enterprises can multiply seed of new varieties from the formal sector to distribute through their local networks (de Boef and Thijssen 2010; Louwaars and de Boef 2012; McGuire and Sperling 2013; Neate and Guei 2010; Rachkara et al. 2017; Samberg et al. 2013). In the case of maize, elements of social network analysis have proven useful for understanding how farmers acquire planting material and related information through direct and indirect network ties, as well as the effects of these relations on seed transaction mode (Badstue 2006). The work of Granovetter and others (Granovetter 1973, 1983, 1985; Haythornthwaite 2002; Smith-Doerr and Powel 2005) on the concepts of strong and weak ties among social actors is particularly relevant to the efforts aiming to strengthen the integration of formal and informal seed systems. Whereas strong ties are associated with intimacy, frequent contact and reciprocal services between close friends, kin or colleagues, weak ties refer to relations of infrequent contact that lack intimacy, such as acquaintances (Granovetter 1973, 1983). We will return to these concepts in the results section to illustrate how farmers use social networks to acquire sweetpotato planting material both within and outside their communities.

This article adds to this broad literature by presenting new insights from Tanzania on smallholder farmers’ practices around the acquisition and distribution of sweetpotato planting material. It examines three specific issues: (i) farmers’ sources of planting material; (ii) factors that influence farmers’ sourcing of planting materials outside their farms; and (iii) the types of transactions and social relations involved in farmers’ acquisition and distribution of sweetpotato planting material. The knowledge generated in this study can inform interventions seeking to strengthen small-scale farmers’ access to improved, high quality sweetpotato germplasm; as well as research and development (R&D) strategies aiming to integrate formal and informal systems for accessing and distributing VPCs planting material.

## 2 Methods

### 2.1 Data collection

The study employed a mixed-methods research approach, integrating both qualitative and quantitative methods, including (1) a structured household survey, (2) in-depth, semistructured interviews with women sweetpotato growers and (3) women-only and mixed focus group discussions (FGDs) with sweetpotato farmers.

The 2010 CIP household survey dataset provided quantitative data for 621 households in nine districts from Tanzania’s Mwanza and Mara regions. The sample households were selected using a stratified probability random sampling technique (Sindi and Wambugu 2012). The structured interviews in the survey followed a predetermined and standardized list of close-ended questions that were asked to all respondents in the same order. The questions covered many areas, including farmer sweetpotato vine sources; farmer practices of conserving planting material during the long dry period; the types and number of sweetpotato vine transactions; farmer knowledge of sweetpotato diseases; farmer perceptions around the quality of planting material; and sales of sweetpotato vines and/or storage roots.

In addition, 28 women sweetpotato growers were interviewed in July 2010 and again in July 2011. The respondents came from three villages: Kitaramaka in the Bunda district of the Mara region, Matale in the Magu district of the Mwanza region, and Nyakanga in the Musoma Rural district of the Mwanza region. The three villages, all of which participated in the Tanzania CIP 2010 household survey, hold contrasting agro-ecological and socio-economic characteristics and conditions. All in-depth interview respondents were women small-scale farmers purposively selected from the CIP 2010 household survey sample.

Finally, six FGDs were carried out in July 2012 in the same three study villages. In each community, FGDs were held with a women-only and a mixed-sex group. With the assistance of local key informants, focus group participants were identified based on the following criteria: marital status (a balance was made to ensure participation of married, widowed and divorced people) and socio-economic status assessed on the basis of housing conditions and cattle ownership. A household was considered to be poor if (i) the roofing material of the house was made of grass and the walls were made of mud and (ii) if the household did not own any cattle. Otherwise, the household was considered well off. For the distribution of focus group participants by gender and village, see Table 1.

**Table 1:** Number of participants in the FGDs and their distribution by gender across all three villages

Study site	Number of participants in the women-only focus group	Number of participants in the mixed-sex focus group		
		Number of women	Number of men	Total number
Kitaramaka	9	5	5	10
Nyakanga	8	5	5	10
Matale	10	5	5	10
Total	27	15	15	30

The FGDs included questions on: sources and practices for acquiring sweetpotato vines; reasons for seeking vines outside own farms; types of transactions used for acquisition/distribution of vines; social relationship involved in vine transactions; the number of plots worked by women; and farmers’ experiences with training in sweetpotato production and management. These questions were followed by several open-ended questions, in which the respondents provided rich, extensive answers.

A process of member checking was systematically carried out to minimize potential bias resulting from time differences in data collection periods between the baseline survey conducted in 2010, in-depth interviews in 2010 and 2011, and FGDs in 2012. The diverse and complementary data collection approaches across the communities helped establish pockets of multilayer information within the general intervention area.

### 2.2 Data analysis

The household survey data was coded in Excel and transferred to STATA for analysis. Descriptive statistics were used to analyze: (i) the characteristics of household heads and number of children in households under study (Table 2); and (ii) farmers’ regular source of sweetpotato vines (Table 3). In addition, binary logistic regression was employed to determine the relationship between farmers’ sourcing of sweetpotato vines (from their own farm or elsewhere) and a number of independent variables related to (i) farmers’ individual characteristics (age, age square, education, education squared, sex, training in sweetpotato production and management, knowledge of sweetpotato diseases and principal or secondary agricultural activity); (ii) household socioeconomic characteristics (household size, total land owned in the 2008/2009 cropping seasons, number of plots worked by a woman household member, access to or ownership of a valley bottom, selling sweetpotato vines or storage roots; farmers’ satisfaction with the quality of planting material present on their farm; and household membership in a crop production association); and (iii) community-level characteristics (wet area, village road type and distance to the nearest outlet market) (Table 4). Lastly, descriptive statistics were also used to analyze farmers’ primary transaction type for sweetpotato vine acquisitions and distributions (Table 5).

**Table 2:** Characteristics of household heads and number of children in households

Variables	All households		Male-headed households		Female-headed households		Mara		Mwanza	
	(*N* = 621)		(*N* = 490)		(*N* = 131)		Region (*N* = 202)		Region (*N* = 419)	
	No.	%	No.	%	No.	%	No.	%	No.	%
Age of household head										
26 to 35 yrs.	51	8.1	47	9.6	4	3	15	7.4	36	8.6
36 to 45 yrs.	182	28.1	148	30.2	34	26	54	26.7	128	30.5
> 45 yrs.	388	61.4	295	60.2	93	71	133	65.8	255	60.9
Education of the household head										
No education/pre school	137	22.1	78	15.9	59	45	31	15.3	106	25.3
Primary	438	70.5	368	75.1	70	53.4	149	73.8	289	69
Secondary	44	7.1	42	8.6	2	1.5	20	9.9	24	5.7
College/University	2	0.3	2	0.4	0	0	2	1	0	0
Number of children in a household										
0 to 2	87	13.9	68	13.9	19	14.5	28	13.9	59	14.1
3 to 5	245	39.4	186	38	59	45	92	45.5	153	36.5
6 to 8	202	32.5	163	33.3	39	29.8	60	29.7	142	33.9
> 9	87	14	73	14.9	14	10.7	22	10.9	65	15.5

**Table 3:** Farmers’ sources of sweetpotato vines

Question posed: Where do you normally obtain sweetpotato vines from?	All households	
	No.	%
Own farm	349	56.2
Male neighbor	20	3.2
Female neighbor	141	22.7
Relatives	12	1.9
Farmer group	2	0.3
Vine multipliers far away	62	9.8
NGO	2	0.3
Farmers along the lakeshore	33	5.3
Total	621	100

**Table 4:** Logistic regression results for farmers sourcing sweetpotato vines from their own farms or elsewhere

Independent variables	Description of the variables	Model capturing all households		
		b	e^b^
I. Individual characteristics				
Age	Age of the person who is knowledgeable about sweetpotatoes (years)	0.081!	1.084
Age squared	Farmers’ age squared	−0.001!	0.999
Education	Number of school years attended by the respondent	0.01	1.01
Education squared	Farmers’ education squared	−0.002	0.998
Sex	Dummy variables for male (1) and female the reference group (0)	0.055	1.057
Training in sweetpotato production and management	Dummy variables for farmers who have received the training (1), and farmers who have not been trained serve as a reference group (0).	0.587!	1.798
Knowledge of sweetpotato diseases	With values 0 to 1.0 if all questions are answered incorrectly, 0.2 if one question answered correctly, 0.4 if two questions answered correctly, 0.60 if 3 questions answered correctly, 0.80 if 4 questions answered correctly and 1 if 5 questions answered correctly	0.598!	1.818
Agriculture as principal or secondary activity	Dummy variables for the categories principal activity (1) and secondary activity (2)	−2.006**	0.135
II. Household characteristics				
Household size	Number of people living in the household (meaning sharing the same kitchen), children and adults of all ages	0.02	1.02
Total land owned in the 2008/2009 cropping seasons (acres)	The size of the land that the farmer owns in the 2008/2009 cropping season in acres. For missing values, the average of the district’s land holding was used to fill in the gap. This is a continuous real variable.	0.012	1.012
Number of plots for which the woman of the household has control over what is grown	The number of plots of land owned by the woman of the household. For missing values, the average of the district’s land holding was used to fill in the gap. This is a continuous real variable.	0.151*	1.162
Access to or ownership of land at the bottom of a valley	Dummy variables for owning or having access to valley bottom (1) and farmers who do not own or do not have access to a valley bottom (0)	0.145	1.156
Selling sweetpotato vines	Dummy variables for selling sweetpotato vines: if yes (1), if no (0)	−0.209	0.812
Selling sweetpotato storage roots	Dummy variables for selling sweetpotato storage roots: if yes (1), if no (0)	0.764***	2.147
Farmer satisfaction with the quality of planting material present on her/his farm	Refers to farmers’ satisfaction with the quality of vines that are usually available at the time of planting. With categories: 1 if satisfied, 2 if somewhat satisfied and/or not satisfied	−0.22	0.803
Household membership in a crop production association	Dummy variables for any member of the household actively participating in any savings, credit, women or farmer association: if yes (1), if no (0)	0.193	1.213
III. Community-level characteristics				
Wet area	Area near the lake. Dummy variables 1 for wet area and 0 otherwise	0.389	1.475
Village road type	The type or road that provides the main access to this village. Dummy variables 1 for foot paths and/or for secondary earth road; 2 for primary earth or murram road and/or for tarmac road; and 3 for water transport.	−0.758***	0.469
Distance to the nearest outlet markets (km)	The distance to the nearest outlet/market to buy seed or fertilizer. This is a real continuous variable represented in km.	0.006	1.006
Constant		−0.626	
Number of sample size (N)		621	
−2 Log Likelihood		73.95	
Model Chi-square		0	
Pseudo R^2^		0.0869	

! *p* < 0.10;*p < 0.05;**p < 0.01;***p < 0.001 and e^b^ = odds ratio

**Table 5:** Main transaction types in farmers’ sweetpotato vine acquisitions and distributions

Type of transaction	No payment (Gift/free)		Payment (purchase/sale)		Total	
	No.	%	No.	%	No.	%
Acquisitions	286	59	196	41	482	100
Distributions	428	84	80	16	508	100

Qualitative data from in-depth interviews and FGDs were transcribed and coded for textual analysis following the procedures outlined by Creswell (2007). The coding and analytical process aided in identifying themes and information used to elaborate, illustrate and clarify results from the quantitative household survey. From the in-depth, semi-structured interviews with women sweetpotato growers, we also analyzed the mode of transaction and social relations involved in household level sweetpotato germplasm acquisitions and distributions. To protect the respondents’ anonymity, all study participant names have been replaced with pseudonyms.

## 3 Results

Of the 621 households in the CIP 2010 household survey, 490 or 79% were male headed and 131 or 21% female headed (Table 2). Household heads were 51 years old on average, with the average age of female heads 54 and of male heads 50. More than one fifth of household heads in the sample had no formal education, and the average level of educational attainment was five years (Table 2). On average, female household heads had three years of education compared to six years for their male counterparts. Average household size was eight people and the average number of children per household five. Residence was predominantly patrilocal and homesteads were situated close to each other.

### 3.1 Smallholder farmers’ sources of sweetpotato vines

Overall, 62.5% of household survey respondents confirmed that they normally try to conserve sweetpotato vines during the long dry period, and the most common way of acquiring planting material is indeed from one’s own farm, as reported by more than half of the respondents (56.2%, see Table 3). The second most common source of vines is from neighbors in the community (25.9%), the vast majority of which are female neighbors (22.7%). The third most common source of sweetpotato vines are vine multipliers located relatively far away from the farmers’ fields (9.8%), followed by farmers along the lakeshore (5.3%), relatives (1.9%), farmer groups (0.3%) and NGOs (0.3%). Table 3 provides an overview of where farmers access sweetpotato vines.

### 3.2 Factors associated with sweetpotato vine acquisition outside a farmers’ own farm

The household survey’s null hypothesis suggested that there is no relationship between farmers seeking sweetpotato vines outside their own farms and individual, household and community-level characteristics. However, the analysis identifies eight factors indicating significant statistical association with farmers’ sweetpotato vine acquisition outside their own farms. These include five individual factors (age, age squared, training in sweetpotato production and management, knowledge of sweetpotato diseases and whether agriculture is the farmer’s principal or secondary activity), two household level factors (number of plots a woman household member has control over what is grown, and selling sweetpotato storage roots), and one community factor (the village road type) (Table 4).

We did not find multi-collinearity in our variables, all of which registered variance inflation factors (VIFs) of less than 2.50. Below we explain in more detail the implications of the independent variables that have a statistically significant impact on the dependent variable vine sourcing at the 5% significance level or below.

**Individual factors** As farmers age, they are more likely to be seed savers than seed seekers and become less able to fully participate in farming activities, a finding that highlights the significance of life cycle effects. Moreover, the results imply that farmers whose main economic activity is agriculture are more likely to be concerned about conserving planting material on their own farm plots than farmers for whom agriculture is their secondary activity. Furthermore, farmers who have received training in sweetpotato production and management are almost twice as likely to produce their own planting material compared to those who have not received any training. Lastly, farmers who have knowledge about sweetpotato diseases are about twice as likely to produce their own planting material as those without such knowledge.

**Household-level factors** The more plots the woman of the household manages, the higher the likelihood that she sources vines from her own farm. In addition, the results indicate that farmers who sell sweetpotato storage roots are twice as likely to produce their own planting material as those who do not sell sweetpotato storage roots.

**Situational/community factor** For farmers residing in villages with a relatively good tarmac road, the odds of sourcing vines from their own farms decreases by almost half, relative to farmers who reside in communities primarily served by foot paths. In other words, farmers from villages that are passable only by footpaths and/or secondary earth roads are more likely to obtain vines from their own farms than farmers who reside in more urbanized villages.

Overall, the findings from the above analysis indicate a relationship between the farmer’s likelihood of seeking vines outside his or her own farm and some of the individual, household and community-level characteristics. The characteristics that appear to make farmers more likely to source vines from their own farm plots include: being an older or experienced farmer; having received training on sweetpotato production and management; having knowledge of sweetpotato diseases; selling sweetpotato storage roots; and being from a household where several farming plots are controlled by the woman of the household. On the other hand, according to the results in Table 4, farmers who treat agriculture as a secondary rather than as a primary activity, and who reside in a village with good/tarmac road infrastructures, are more likely to source vines from outside their own farm plots.

Our findings show that saving or producing planting material on one’s own farm is the most common practice in the study area. There are, however, occasions when farmers have to acquire sweetpotato planting material outside of their own farms—for example, when the plants intended for planting material have succumbed to drought thus leaving the farmer with no, or insufficient, planting material; or when farmers are keen to try out a new variety (out of curiosity) or wish to recuperate a lost one. In what follows, we take a closer look at how vine transactions take place in some of these situations.

### 3.3 Types of transactions and social relations in farmers’ acquisitions and distributions of sweetpotato planting material

The household survey instrument only distinguished between two modalities for sweetpotato planting material transactions: (1) receiving/giving vines in return for payment (purchase/sale) and (2) receiving/giving vines without payment in return (i.e., as a gift or free). The data analysis makes clear that the most predominant type of transaction was that of gift, both for farmers who distributed vines (84%) and for those who received planting materials from others (59%) (Table 5). Meanwhile, the exchange of payment in return for vines, or purchase/sale, was used in 16%of vine distributions, and 41% of the vine acquisitions reported in the 2010 household survey.

The DVMs produce vines in large quantities for the purpose of selling them to other farmers. However, most of the CIP 2010 household survey data participants indicated that they did not source vines from DVMs; only 2% of respondents (10 out of the 508) who gave out or sold vines were vine multipliers.

### 3.3 Types of transactions and social relations in farmers’ acquisitions and distributions of sweetpotato planting material

The household survey instrument only distinguished between two modalities for sweetpotato planting material transactions: (1) receiving/giving vines in return for payment (purchase/sale) and (2) receiving/giving vines without payment in return (i.e., as a gift or free). The data analysis makes clear that the most predominant type of transaction was that of gift, both for farmers who distributed vines (84%) and for those who received planting materials from others (59%) (Table 5). Meanwhile, the exchange of payment in return for vines, or purchase/sale, was used in 16%of vine distributions, and 41% of the vine acquisitions reported in the 2010 household survey.

The DVMs produce vines in large quantities for the purpose of selling them to other farmers. However, most of the CIP 2010 household survey data participants indicated that they did not source vines from DVMs; only 2% of respondents (10 out of the 508) who gave out or sold vines were vine multipliers.

Contrary to the household survey, the in-depth semi-structured interviews and FGDs used open-ended questions, and in the data analysis seven different modalities or types of transactions were identified for farmers’ acquisition and distribution of sweetpotato planting material: gift/free, purchase, inheritance, exchange, barter, labor for vines and vine poaching. The qualitative approach produced additional insights into the factors that influence informal sweetpotato planting material transactions.

As already indicated in the household survey results, the most common modality for farmer-to-farmer vine transactions was that of receiving vines as a gift/for free, i.e., without a cash or in-kind payment in return. The four major reasons given by farmers to explain why one would distribute or receive vines for free included: i) the individual asking for vines is someone with whom one has a close social relationship, e.g., a neighbor, a friend or relative—in other words, a strong tie; ii) reciprocity, i.e., returning a favor previously received, or helping out because on another occasion one might need a favor oneself; iii) out of concern for other individuals in the community; iv) casual attitude (it is not a big deal, e.g., when the provider has plenty of vines for no other use). In line with the notion of economic relationships as embedded in other social institutions (Polanyi 1977), it is noticeable that the first three reasons reflect different variations of a social system with strong value on social obligations, a core element in many largely subsistence-based economies.

*Purchase*, or the use of money in return for vines, is a form of transaction that appears to be less dependent on the social relationship or level of social obligations expected between the parties involved. Study participants noted that this type of transaction can involve any two parties, including strangers and acquaintances or weak ties. Data from individual interviews and FGDs revealed three main motives for a monetary transaction: i) to obtain money, ii) not having any social or kinship relations with the vine seeker and therefore no moral obligation to give for free and iii) paying for vines as a sign of appreciation (when acquiring vines). An in-depth interview with Salima, leader of the *Uongozi* group (one of the DVMs under the *Marando Bora* project), exemplifies the challenge of selling vines to people from her own small community. Salima started the business of producing sweetpotato vines in early 2011, but only sold vines to farmers from other communities. As she explained about people from her own village, “When they hear you are selling, they do not understand. People from the village also come to get vines, but they begask for them for free, whereas people from elsewhere, strangers, know they are strangers and therefore are prepared to pay.” However, the following year, Salima reported that now some farmers in her community understood that she was trying to run a business and paid for vines. The example illustrates how social relations and related moral obligations influence the type of transaction, and demonstrates the need for emerging commercial vine multipliers to be businessoriented and able to explain their endeavor to community members.

Gifts and purchases accounted for the vast majority of the transactions recorded. In the following, we briefly describe the other types of transactions identified in the in-depth interviews and FGDs, before turning to the role of weak and strong ties in the case of two specific sweetpotato farmers.

Children, foster children and daughters-in-law often obtain sweetpotato planting material through inheritance. This can happen while the parents are living, when the children become more independent of their parents and start to farm on their own, or when the parents pass on. In most cases, farmers who obtained vines through inheritance received them from their husbands’ parents upon marriage.

Farmers sometimes *exchange* sweetpotato vines of one kind for the same or a different quantity of vines of another variety. The transaction does not involve cash and happens at both parties’ convenience. A different variation of this is *bartering*, where sweetpotato vines are given in return for some other in-kind good of use to the vine provider, for instance, beans, *udaga* (dried cassava flour) or maize seeds. Bartering is most common in communities where the agroecological conditions tend to challenge farmers’ own conservation of planting material. This is for example the case in Suzana’s village, Matale. Suzana traveled to the village of Nemba, a wetland in the Lake Zone region known as a place where farmers always have vines. Being poor, she did not have money to pay for the vines and instead negotiated with the vine provider to receive sweetpotato vines in exchange for maize. Yet another variation of exchange is *labor for vines*, when vines are obtained in return for work. This is the least common of all the vine transactions previously noted. One of the informants, Angelina from Nyakanga, described her experience with this, “I was hired by Mama Wambura to plant sweetpotato vines and at the end of the job, she gave me *marando*.” As the examples show, these modalities can serve as alternatives for those who are not able to pay cash in return for planting material.

Finally, in-depth interview respondents and FGD participants in all three communities lamented that it is not unusual for people to take sweetpotato planting material from other farmers’ plots without seeking permission. We refer to this as *vine poaching*. Zawadi from *Nyakanga* bravely shared the following, “When I first came to the village, I acquired my first batch of *marando* from the farms that are located next to my plot, without asking permission from the owners.” Since then, she herself has also been exposed to vine poaching. Farmers mentioned farm location (i.e., being far from the homestead) and the general perception of the low value of sweetpotato planting material, as compared to seeds of other crops, such as maize, as two main issues related to this. On the other hand, some farmers feel that there are so many *marandos* that vine poaching is not a matter to be taken seriously. However, Maria from *Matale*, where the supply of vines is often lacking at the time of planting, disagreed, “It is not right for someone to cut [vines] on someone else’s farm. One has to pay.”

### 3.4 The strength of weak ties and the role of strong ties: The cases of Ana and Salome

**Fig. 1:**
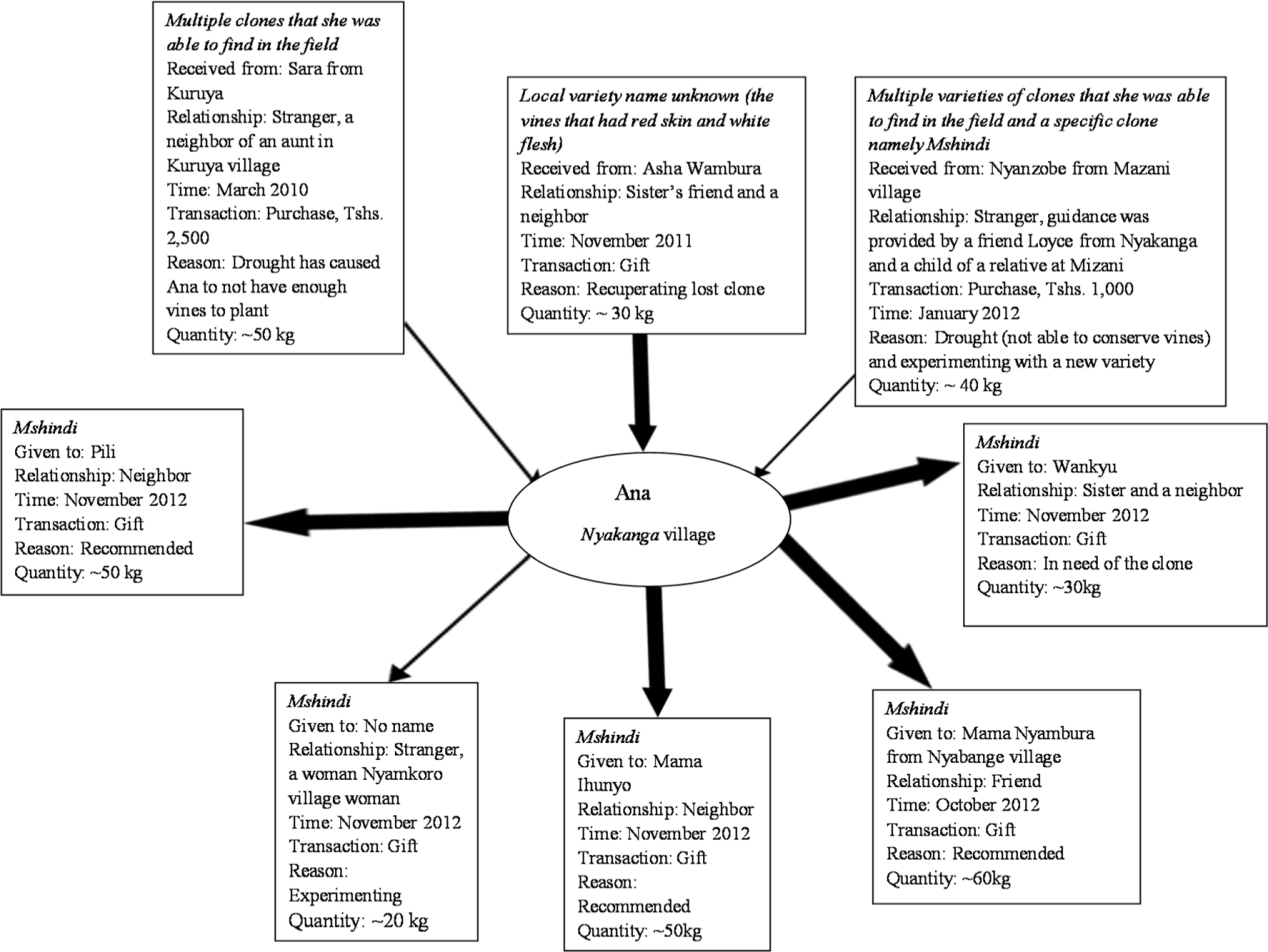
Vine flow diagram, Ana

**Fig. 2:**
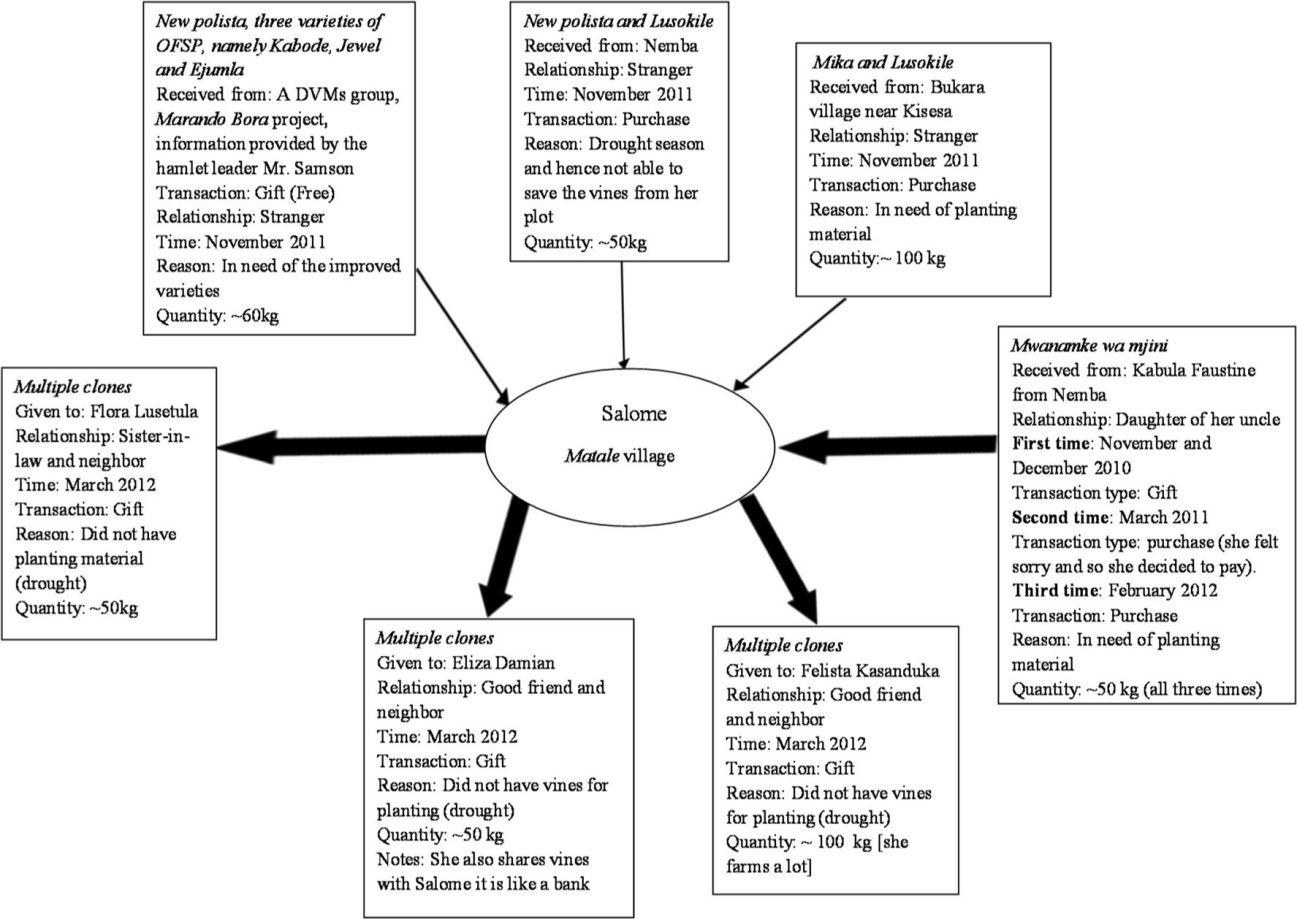
Vine flow diagram, Salome

To further illustrate the complexities in farmer-to-farmer sweetpotato vine transactions, we present a flow-chart analysis of planting material transactions of two female farmers, Ana Kanyagia from Nyakanga village (Fig. 1) and Salome Kapinga from Matale village (Fig. 2). The two flow diagrams represent the networks of vine exchanges among farmers, including the social relations involved, modes of transaction, reasons for acquiring the vines, quantity of vines acquired, time of vine acquisition for each transaction and in some cases the type of sweetpotato germplasm received. The bold arrows symbolize “strong tie” meaning close social relationships (e.g., friends and family) between vine providers and vine receivers, and the thin arrows symbolize “weak ties”, meaning a distant social relationship (e.g., stranger).

Ana is a 28-year-old farmer and single mother of one child. She lives in the family homestead with her mother, who is a widow, and for whose care Ana is responsible for as the youngest child. An active farmer, Ana’s secondary occupation is tailoring clothes. Figure 1 shows the incoming and outgoing flows of sweetpotato planting material from Ana’s household, as reported during the in-depth interview.

Figure 1 shows that in most cases, Ana’s vine transactions take place directly between two actors. In several cases, the relationship between Ana and the vine receiver or provider is multi-stranded, meaning that in addition to being involved in mutual seed exchange, they are also neighbors, kin or friends. Ana for instance provided vines of the *Mshindi*^3^ variety to her sister, who is also her neighbor. Furthermore, in all cases in which strong ties exist, money is *not* used as a mode of transaction. In contrast, with the exception of one case, vines are paid for with money when ties are weak. Sometimes farmers won’t share planting material with people they do not perceive as serious or competent farmers (e.g., Badstue 2006). When Ana wanted to acquire vines of the *Mshindi* variety, she approached Loyce, a woman farmer in the village known for growing *Mshindi*.

Ana and Loyce did not know each other beforehand, and according to Ana, Loyce thought Ana was lazy and not a serious farmer. Instead of selling vines to Ana, Loyce told her to go all the way to Mazani village, where Ana was to ask for a relative and friend of Loyce named Nyanzobe, who would sell her *Mshindi* vines. Ana eventually traveled to Mazani to buy *Mshindi* vines from Nyanzobe (weak tie), and she has since distributed *Mshindi* vines to several other farmers in her own village (strong ties) and beyond.

Salome is a 48-year-old farmer and mother of five. With her husband seriously ill, Salome is her household’s de facto head. Although farming is Salome’s only economic activity, she is active in her community and heads her village’s local women’s self-help group. Figure 2 shows the incoming and outgoing sweetpotato vine flows from Salome’s household. Like Ana, most of Salome’s vine transactions occur directly between two actors, and multi-stranded relationships are observed in relation to several of the transactions. Similarly, gift was the most common type of transaction when conducted with strong ties. However, in the case of the *Mwanamke wa mjini* (local) variety transaction with her niece Kabula, Salome explained that she gave her niece money in return for the vines on two of the three occasions because she “felt sorry”.

In addition to her local community involvement, Salome is a member of the HISA^4^ credit and savings group in another village. On one occasion, she learnt from Mr. Samson, a fellow member of the HISA group and hamlet leader in the other village, about an opportunity to access high quality germplasm of improved sweetpotato varieties from a DVM group under the *Marando Bora* project. Through her connection to Mr. Samson (weak tie), Salome managed to qualify for provision of quality planting material of New Polista and three improved OFSP varieties: *Kabode, Jewel* and *Ejumula*. Meanwhile, farmers from Matale village not in the HISA group did not receive this information or benefit from the opportunity. This example shows that weak ties can also be important; in this case, Mr. Samson exercised his influence to put a word in for Salome so that she could receive quality planting material.

## 4 Discussion

More than half of the small-scale farmers in the study area obtained all or most of their sweetpotato planting material from their own farms. Those who did not succeed in this, or who were not accustomed to conserving vines during the dry season, mainly obtained their planting material from other local farmers. According to the statistical analysis, older or more experienced farmers; farmers who were trained in sweetpotato production or have knowledge of sweetpotato diseases; farmers who sell sweetpotato storage roots; and farmers from households where the woman manages several plots, were more likely to obtain sweetpotato planting material from their own farms. In contrast, farmers from communities served by good/tarmac road infrastructure, and those for whom agriculture was a secondary activity, were more likely to acquire vines from others. In other words, the more knowledge about and experience with sweetpotato production farmers have, the more focus farmers place on agriculture as a livelihood strategy, and the more engaged farmers are in the sale of sweetpotato products, the *less* likely they will be to acquire sweetpotato planting material from other sources.

A core element of the sweetpotato seed system in the study area is a self-sustaining dynamic, whereby most farmers produce their own planting material. If, or when, this fails, they rely on strong ties with other farmers through whom they acquire sweetpotato planting material on favorable terms, typically without having to pay. Providing planting material to others in this way forms part of the mutual exchange of favors between kin, friends and other community members, which makes life possible in poor and partly subsistence-based rural contexts. The local system for acquisition of sweetpotato planting material should therefore be understood as embedded in a social system of reciprocity, mutual support and social obligations. The vast majority of the vine transactions in the study communities took place as gifts, or for free, and the use of cash payment in return for vines was but the second most common transaction type (Table 5). As the qualitative data shows, other modalities for acquiring vines from other farmers without the use of money as payment exist, although they are less common. Nevertheless, in this socially embedded system the alternative transaction modalities (e.g., bartering or vines in return for labor) make it possible to acquire sweetpotato planting material, even for those who may not be able to pay for it with money. In this type of context, the introduction of a commercially based model for sweetpotato planting material provision would face challenges, as also clearly demonstrated above in the experience of Salima from the Uongozi DVM group under the *Marando Bora* project.

At the same time, some farmers are curious and interested in trying out new things, and when opportunities present themselves (e.g., free samples of improved planting material), many farmers take advantage and plant it to see how it performs, as in the case of Salome who acquired quality planting material of four improved varieties via her acquaintance with Mr. Samson. Similarly, when in search of a particular kind of planting material, some farmers are willing to go far. When Ana acquired the *Mshindi* variety, she was short of planting material but could probably have acquired the amount of vines she needed through close social relations (strong ties). However—determined to try out the *Mshindi* variety and prepared to pay for it—she eventually traveled to Mazani and obtained it from a stranger (weak tie). Both Ana and Salome obtained exotic sweetpotato material through weak ties, and subsequently, shared these same materials with other farmers with whom they had strong ties, without demanding payment in return.

As these examples illustrate, Granovetter’s (1983) notion of “the strength of weak ties”, is useful to understanding informal seed systems and the challenges and opportunities related to the integration of formal and informal seed systems. In fact, as we have seen, a closer examination of the ways farmers in the study area traditionally source sweetpotato planting material reveals an informal, socially embedded system capable of reproducing and distributing sweetpotato planting material at very low cost. Under these circumstances, a feasible way of enhancing local farmers’ access to improved sweetpotato materials would seem to be through subsidized targeted distribution of improved variety planting material to local farmers known to be vine providers and knowledgeable about sweetpotato cultivation. Via the complementarity of strong and weak ties, this approach could strengthen farmers’ access to improved germplasm through existing local channels, while limiting the level of external investment needed for multiplication and free distribution of germplasm at regular intervals, i.e., every few years. While local dynamics for acquisition and provision of planting material would facilitate diffusion of the new improved varieties to other farmers, over time the quality of the planting material would be expected to degrade due to virus pressure and other disease and pest factors, hence the need for renewed influxes of quality germplasm every few years. To mitigate the problem of virus affecting the production and quality of planting material, the development and diffusion of virus-resistant varieties would have strategic importance.

Among the other dimensions to take into account when exploring ways to strengthen systems for provisioning of sweetpotato planting material is the overall goal of intervention. For example, if the objective is to improve food security and nutrition (e.g., Vitamin A intake through production and consumption of OFSPs), the subsidized targeted planting material distribution, or infusion approach, may be a justifiable and realistic option, where the local system for acquiring sweetpotato planting material is embedded in a social system of reciprocity and mutual help, as in the study area presented here. The low value generally associated with sweetpotato vines, and the level of market access and capacity, are other factors to consider. If market access and demand for sweetpotato roots is limited, subsidized infusion of improved varieties into the local system via local community organizations and locally recognized sweetpotato knowledge holders and vine providers may be the most feasible approach. However, where there are large or growing sweetpotato market opportunities for many smallholder producers; or regional NGO demand for sweetpotato planting material in bulk for input supply in community-level interventions, there may be a market for sweetpotato vines and hence potentially suitable conditions for support of a commercial, mainly informal system for provisioning of sweetpotato planting material. Similarly, where agriculture and sweetpotato production is a secondary or side activity for many (e.g., farmers near urban centers who depend mostly on off-farm income opportunities or who focus on a different crop portfolio), a commercially based system for acquisition/provision of sweetpotato planting material may have better prospects, as indicated by the finding that farmers from communities with good/tarmac road infrastructure, and those for whom agriculture is a secondary activity, are more likely to acquire vines from others (Table 4), and presumably, to pay for them, an assumption which would require testing.

The question of sustainability is often raised when justifying interventions. In the study presented here, we observe a socially embedded informal system for acquiring sweetpotato planting material, which appears to be relatively well functioning in terms of its ability to maintain specific sweetpotato materials over prolonged periods of time and ensure supply of planting material to local farmers at low cost. As such, the system itself can be considered relatively sustainable, compared to the typical project model as referred to by Gibson (2013) that depends on external technical and financial support to establish and maintain a separate system for multiplication and distribution of vines. One can hypothesize that even with an initial subsidized infusion of high-quality planting material of improved sweetpotato varieties, repeated every few years, this approach for enhancing smallholders’ access to improved sweetpotato varieties would be more sustainable and feasible compared to the more traditional project model (ibid.).

In conclusion, our findings support the call for making use of or building on the existing informal seed system when seeking to strengthen the diffusion and adoption of improved sweetpotato varieties, as suggested by others (Rachkara et al. 2017; McGuire and Sperling 2013). However, when deciding how to proceed, a thorough understanding of the dynamics of the existing system, including information about local key knowledge holders and distributors of planting material, is needed. The aspects discussed in this section are topics to be considered for further research.

## Footnotes

1 The *Marando Bora* Project was a component of the Sweetpotato Action for Security and Health in Africa (SASHA) program, funded by the Bill & Melinda Gates Foundation.

2 In the term “seed system”, “seed” refers to that which is planted to propagate the crop; it does not imply its true botanical meaning.

3 Mshindi is the clone that is favored by all the farmers who sought vines from Ana. The Mshindi’s positive attributes include its short growing cycle; its sweet, sugary and floury quality; its taste; and its ability to produce many storage roots.

4 HISA is a Swahili word, which means shares.
